# Effect of Leaving Centrosymmetric Character on Spectral Properties in Mono-, Bi-, and Triphotonic Absorption Spectroscopies

**DOI:** 10.1021/acsomega.4c06922

**Published:** 2024-09-25

**Authors:** Ricard Gelabert, Miquel Moreno, José M. Lluch

**Affiliations:** † Departament de Química, Universitat Autònoma de Barcelona, 08193 Bellaterra, Barcelona, Spain; ‡ Institut de Biotecnologia i de Biomedicina, Universitat Autònoma de Barcelona, 08193 Bellaterra, Barcelona, Spain

## Abstract

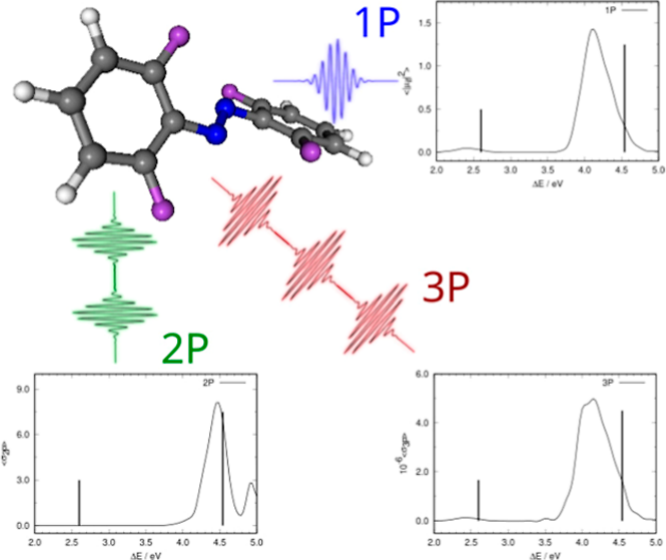

Numerical simulations of the absorption bands of photoswitch *E*-*o*-tetrafluoroazobenzene in DMSO solution under one-, two-, and three-photon absorption conditions combined with the analysis of the behavior of transition probability under distortion of planarity reveal many similarities between the mono- and triphoton spectroscopic behaviors with a two-photon spectrum being set apart. The position of the absorption peak for the studied nπ* and ππ* transitions appears shifted to lower energies (longer wavelengths) than the conventional estimate based on vertical excitation from the ground-state potential energy minimum.

## 1 Introduction

Photoswitches are molecular devices that exist in at least two forms whose interconversion (usually an *E-Z* isomerization) is triggered by the absorption of light with adequate energy.^1,2^ Because the interconversion (or “switching”) takes place with temporal and spatial precision, photoswitches have become essential workhorses in fields like photopharmacology and neuroscience: when cleverly linked to membrane protein receptors or pharmacologically active molecules, light can be used to activate the photoswitch to stimulate individual neurons or activate a drug only where it is supposed to act, in this way minimizing its side effects.^2−10^

One of the challenges faced when designing photoswitches for use in living tissue is ensuring that activation takes place inside the bio-optical window (roughly 600–1000 nm) where the mammal tissue is most transparent to light.^11−13^ While activation with monophotonic absorption (1PA) is technologically simple and convenient, most photoswitches have absorption maxima at much shorter wavelengths, a circumstance that forces irradiation only of the low-energy tails of their absorption bands as these enter the bio-optical window.^14,15^ Not working at (or close to) the absorption band maximum has an impact on the photoconversion rate and the performance of the photocycle in general. A roundabout to this problem exists if multiphotonic absorption (*n*PA) is considered. Under intense illumination, it might be possible to induce the absorption of two or more photons (*n*) at once. In this way, the same energy is split into *n* identical packets, roughly multiplying the required radiation wavelength by *n*. While the technical requirements to induce these transitions are more demanding, they effectively circumvent the problem of activation in living tissue with light of harmless wavelength.^16,17^

In contrast to 1PA, *n*PA with *n* > 1 have been less studied and the factors that determine whether a multiphotonic transition will be intense or not remain more diffuse.^18−20^ Theoretical studies indicate that for centrosymmetric molecules transitions involving the same parity of photons (i.e., *n* being an odd or even number) are related among themselves.^21^ Hence, a molecule having an intense transition in 1PA should also display a strong 3PA to the same excited state, which could be taken as a design parameter. The centrosymmetric property of the isolated molecule will not, however, be preserved when in solution, and it is unclear how much impact this might have in this rule. It is also unknown what to expect for noncentrosymmetric molecules.

It is a standard procedure to estimate the absorption maximum as the vertical transition from the ground-state minimum by virtue of the Franck–Condon principle. Even in the gas phase, this approach can only provide an estimate as to the position of the band’s maximum, but the neglect of vibronic effects (transition between vibrational states in the ground and excited electronic states) can have a noticeable effect on the band maximum and makes it impossible to predict the band shape. This is especially true when the substrate molecule is in solution. In such an environment, a large variety of structures for the molecule exist due to the dynamics. The ground-state minimum is underrepresented in this set, and generally, a broad absorption band is detected.^22−31^ This enables excitation of the substrate at a wavelength noticeably displaced from the one corresponding to the vertical excitation from the ground-state minimum with a certain efficiency. We have recently used this approach to explain the possibility to excite a few photoswitches by irradiation at the low-energy tail of the absorption band.^31^

The question remains as to the effect that solvation will have on the *n*PA spectra, whether the likeness among the odd or even order of the transition stays and how much is the effect of shifting from the vertical transition from the ground-state minimum to the actual *n*PA absorption maximum. The current need for efficiently switchable photoswitches and the interest in *n*PA as a means of activation make this a relevant question. We focus in this work on the photoswitch *E*-*o*-tetrafluoroazobenzene to explore the effects on the absorption maximum in 1PA, 2PA, and 3PA spectra in solution (DMSO). This photoswitch presents two transitions in 1PA: a weak band which experimentally is found with a maximum around ∼458 nm (2.71 eV) described as a nπ* excitation and a more intense band at ∼305 nm (4.07 eV) described as a ππ* excitation.^32,33^ However, this molecule is not planar, and strictly speaking, it lacks an inversion center, so there is no clear-cut distinction between σ and π molecular orbitals. These labels have to be taken as indicative of the correlation of the MOs between which the excitation takes place with respect to those same MOs if the molecule were planar.

## 2 Methods

### 2.1 Single Molecule Optimization and Vertical Transition Calculation

Geometry optimization was performed using the Gaussian16 software suite.^34^ Density functional theory (DFT) was used to determine electronic energies, using the long-range corrected functional CAM-B3LYP, which has been described in recent benchmarks as adequate to describe excitation energies.^35−37^ Optimizations have been done using the 6-311+G(d,p) triple-ζ basis set^38,39^ and, where indicated, in the presence of solvent (DMSO) described by means of the polarizable continuum model (CPCM).^40,41^ The structure found in this way was used in the ensuing vertical transition calculations.

The Dalton software suite was used to compute vertical excitation energies and magnitudes related to the transition probabilities in mono-, bi-, and triphoton absorption (1PA, 2PA, and 3PA, respectively).^42,43^ 3PA calculations represent a challenge because their cost increases very quickly with basis set size, this being the consequence of having to compute the 6 second-order perturbed density matrices besides the three first-order perturbed densities. Besides, current implementation in the Dalton program cannot carry out these 3PA calculations with a polarizable continuum representing the solvent. Because the goal is to compute 1PA, 2PA, and 3PA parameters on a large set of molecular structures, we have explored the effects of using a smaller basis set and of computing the excitations in the gas phase (see the Supporting Information). The double-ζ 6-31G(d) basis set was used to this end.^44−46^ The effect of reducing the basis set size and ignoring the solvent in the excitation energies is small enough as to make this approach the choice for the vertical transitions, as well as for the simulation of the spectral line shape. We note that computation of 3PA cross sections including solvent as polarizable continuum is possible using external software libraries.^47,48^

### 2.2 Simulation of Spectral Lineshapes

To compute the spectral line shape, we have used what is known as the ensemble method: prepare a suitable molecular dynamics simulation of the solute and solvent, select a certain number of snapshots such that correlation among the snapshots is inexistent, and then compute on this ensemble of structures vertical excitation calculations using 1PA, 2PA, and 3PA methodology. This approach has been used in the past by several research groups successfully.^22−31^

#### 2.2.1 Molecular Dynamics Simulations

One solvent molecule (DMSO) was optimized using the procedure detailed above for the optimizations. The program AnteChamber (part of the Amber 2021 software suite)^49^ was used to derive partial charges and to determine the generalized amber force field version 2 (GAFF2)^50^ set of parameters for this molecule. Then, a capped octahedron containing 2000 solvent molecules was constructed and subjected to a protocol of equilibration, including 1 ns of simulation at constant temperature (300 K) and pressure (1 bar) under periodic boundary conditions (PBCs). Mean energy, temperature, and density of the cell were monitored to verify that the values were stable and free of drift. At the end of the process, the density of 1.12 g·cm^–3^ was determined, which compares well to the experiment (1.10 g·cm^–3^).

Simulation of the solvated *E*-*o*-tetrafluoroazobenzene was done within a quantum-mechanical/molecular-mechanics (QM/MM) framework, where the solvent was described using a MM description (GAFF2 parameters determined as described above) and the solute using a QM description (in this case, the density functional with tight binding, DFTB,^51^ with the general-purpose DFTB3 parameter set appropriate for organic molecules).^52,53^ Coupling between the QM and MM parts of the system was introduced by using the electronic embedding scheme under PBC. A single solute molecule was solvated using the solvent box and subjected to a short 1.5 ns equilibration run at a constant pressure (1 bar) and temperature (300 K). The values of temperature, energy, and density were monitored to verify the mean values were stable. After this equilibration phase, the system was considered to be equilibrated. A production run of 30 ns of QM/MM simulation was afterward computed.

#### 2.2.2 *n*PA Spectra Simulation

To assemble a set of representative structures of the system, a snapshot of the MD simulation was sampled every 10 ps (a total of 3000 snapshots) to ensure that no correlation was present in the set. As the effect of the solvent is small in the excitation energies, each snapshot was reduced to the structure of the solute molecule and then three single-point vacuum calculations were done at the DFT level of theory (with the CAM-B3LYP functional) and 6-31G(d) basis set to determine the excitation energies of the 10 lowest-lying excited states, as well as the transition dipole moment (μ_*fi*_), 2PA and 3PA absorption cross sections: σ_2_ and σ_3_. As the square modulus of the transition dipole moment (|μ_*fi*_|^2^) is proportional to the 1P transition probability, these three magnitudes are taken to be in all cases proportional to the transition probability using one-, two-, and three-photon absorption between two states.

It is possible to build up a simulated one-photon absorption spectrum line shape at 300 K by collection of a series of snapshots of an MD simulation at that temperature and then assembling a list of excitation energies and |μ_*fi*_|^2^ values computed on these snapshots. One can then compute a histogram of excitation energies where each snapshot contributes its value of |μ_*fi*_|^2^ (instead of one, as in a standard histogram). The histogram derived in this way is proportional to the transition probability at each excitation energy and can be taken as a simulation of the relative 1PA spectrum. Operating accordingly with σ_2_ and σ_3_, one obtains the simulation of the 2PA and 3PA spectra, respectively. Note that in the last two cases, the spectrum is computed against the excitation energies, not the energy of the photons triggering the transition (which would be half and one-third the excitation energy value, respectively). A fwhm value of 0.1 eV was used to compute the absorption cross sections.

While computation of 1PA absorptions is very affordable, the costs when considering 2PA and especially 3PA rapidly escalate. On the same computer architecture, the CPU times required to compute 1PA/2PA/3PA are in the ratios 1.0:3.5:58.7. In our laboratory, a single 1PA calculation would take about 13 CPU minutes, which rises to 48 CPU minutes for 2PA and escalates to 13.5 CPU hours for 3PA. These figures are per structure: as the results obtained require the calculation of a set of 3000 structures, this justifies the approach taken in terms of costs involved and accuracy.

## 3 Results and Discussion

Just how sensitive the spectroscopic properties of this molecule are to geometric distortion is already an interesting piece of information for the analysis we intend to do. We have optimized the structure of *E*-*o*-tetrafluoroazobenzene in the gas phase, enforcing planarity. Under this constraint, this molecule has *C*_2*h*_ symmetry and has inversion symmetry. We have then computed a few structures derived from the latter, where the dihedral angle controlling the *E-Z* isomerization is slightly altered, which in fact lifts the centrosymmetric character of this structure. For all these structures, the excitation energies for the S_0_ → S_1_ (nπ*) and S_0_ → S_2_ (ππ*) transitions, along with the corresponding magnitudes related to the transition probabilities under 1PA (|μ_*if*_|^2^, squared modulus of the transition dipole moment), 2PA and 3PA (σ_2_ and σ_3_, commonly known as absorption cross sections) spectroscopies have been calculated. Results are listed in Table 1.

**Table 1:** Vertical Transitions from Geometries of *E*-*o*-Tetrafluoroazobenzene Close to Planarity

	S_0_ → S_1_ (nπ*)				S_0_ → S_2_ (ππ*)			
δ_EZ_^a^	Δ*E*	|μ_*if*_|^2^	σ_2_	σ_3_	Δ*E*	|μ_*if*_|^2^	σ_2_	σ_3_
deg	eV	a.u^b^	GM^c^	a.u^d^	eV	a.u^b^	GM^c^	a.u^d^
180	2.46	0	8.1 × 10^–3^	3.9 × 10^–1^	4.26	7.7 × 10^2^	8.8 × 10^–8^	2.3 × 10^7^
170	2.41	8.6 × 10^–3^	6.9 × 10^–3^	2.1 × 10^5^	4.27	7.5 × 10^2^	5.0 × 10^–2^	2.3 × 10^7^
160	2.27	1.0 × 10^–1^	4.2 × 10^–3^	6.5 × 10^5^	4.28	6.9 × 10^2^	5.8 × 10^–1^	2.3 × 10^7^

a δ_EZ_ corresponds to the dihedral angle formed by atoms C_A1_–N_A_–N_B_–C_B1_ (see Figure 1).

b 1 au for |μ_*if*_|^2^ = 7.19 × 10^–59^ C^2^ m^2^ = 6.45 D^2^.

c 1 GM (Goeppert–Mayer) = 10^–50^ cm^4^ s photon^–1^.

d 1 au for σ_3_ = 1.28 × 10^–83^ cm^6^ s^2^ photon^–2^.

It is reasonable to expect that a continuous transformation leading from the centrosymmetric structure and away from planarity (hence, eliminating the inversion center) should induce a continuous change in the transition probabilities. In this way, it should be expected that forbidden transitions when the molecule is centrosymmetric will probably be allowed when the inversion center is eliminated but remain weak nonetheless. The nπ* excitation is forbidden under 1PA conditions when the inversion center is present. However, even small deviations from planarity have a remarkable effect, and 1P transition probabilities increase rapidly. This is actually what makes exciting the nπ* transition a feasible possibility to photoisomerize this photoswitch under 1PA conditions as at any temperature the fraction of molecules that retain the inversion center is negligible.^31^ Aside from this, a remarkable fact arises from this preliminary exploration: 1PA and 3PA spectroscopies are predicted to behave in a very similar way to each other in both the nπ* and ππ* excitations, with 2PA clearly showing a distinct behavior. Moreover, the behavior detected in the nπ* excitation is opposite the one seen in the ππ* excitation. For the nπ* excitation, 1PA and 3PA transition probabilities increase clearly when the structure leaves planarity, while at the same time, 2PA transition probability decreases slightly. Conversely, for the ππ* excitation, 1PA and 3PA transition probabilities remain approximately constant or decrease slightly, while 2PA increases substantially when leaving planarity.

The observed behavior of the transition probabilities when leaving planarity (losing centrosymmetric character) can be in part and qualitatively rationalized as follows:

- Transition to S_2_: excited state S_2_ is described as the result of a ππ* excitation and is of *B*_*u*_ symmetry. Transition via 2PA is symmetry forbidden, which means that lifting centrosymmetry will lead to an increase of the 2PA cross section. Transitions via 1PA and 3PA are, in contrast, allowed. In these cases, being the orbitals involved in the excitation of π character the largest overlap will arise precisely when the molecule is planar and will decrease as torsion sets in. Consequently, a decrease of transition probability is expected when the molecule loses planarity in 1PA and 3PA. This is what is actually observed in Table 1.
- Transition to S_1_: excited state S_1_ is described as the result of an nπ* excitation and is of *B*_*g*_ symmetry. 1PA transition is symmetry forbidden, and transition probability is very small for 3PA. Torsion out of plane and elimination of the center of symmetry causes a small dipole moment to arise, and as a result, 1PA transition is no longer forbidden and will increase as torsion sets in. 3PA cross section will follow the same trend as 3PA cross section is proportional to a power of the transition dipole moment when permanent dipole moment is zero (in planarity). Thus, it is suggested that abandonment of the planarity will cause 1PA and 3PA cross sections to increase. This is actually what is seen in Table 1.

Finally, the 2PA transition to S_1_ is not symmetry forbidden. The value of the cross section in this case decreases slightly. With the information at our disposal, we are unable to trace this behavior to a structural cause.

The structure of *E*-*o*-tetrafluoroazobenzene was optimized in DMSO (using a continuum solvation model). This molecule has inversion symmetry only when it is in a planar conformation. The absolute minimum of the molecule has a staggered conformation due to the steric interaction of the fluorine atoms on both phenyl rings. Figure 1 shows the actual arrangement of the two phenyl rings as well as the atom labels that will be used in the forthcoming discussion. We define δ_EZ_ as the dihedral angle formed by atoms C_A1_–N_A_–N_B_–C_B1_ and δ_PA_ and δ_PB_ as dihedrals C_A2_–C_A1_–N_A_–N_B_ and C_B2_–C_B1_–N_B_–N_A_. δ_EZ_ defines the *E-Z* isomerization state, while δ_PA_ and δ_PB_ control the coplanarity of the N=N moiety with phenyl rings A and B, respectively. The minimum has δ_EZ_ = 177°, and δ_PA_ = δ_PB_ = −31°. The minimum energy structure has *C*_2_ symmetry, so that it has no inversion center.

**Figure 1:**
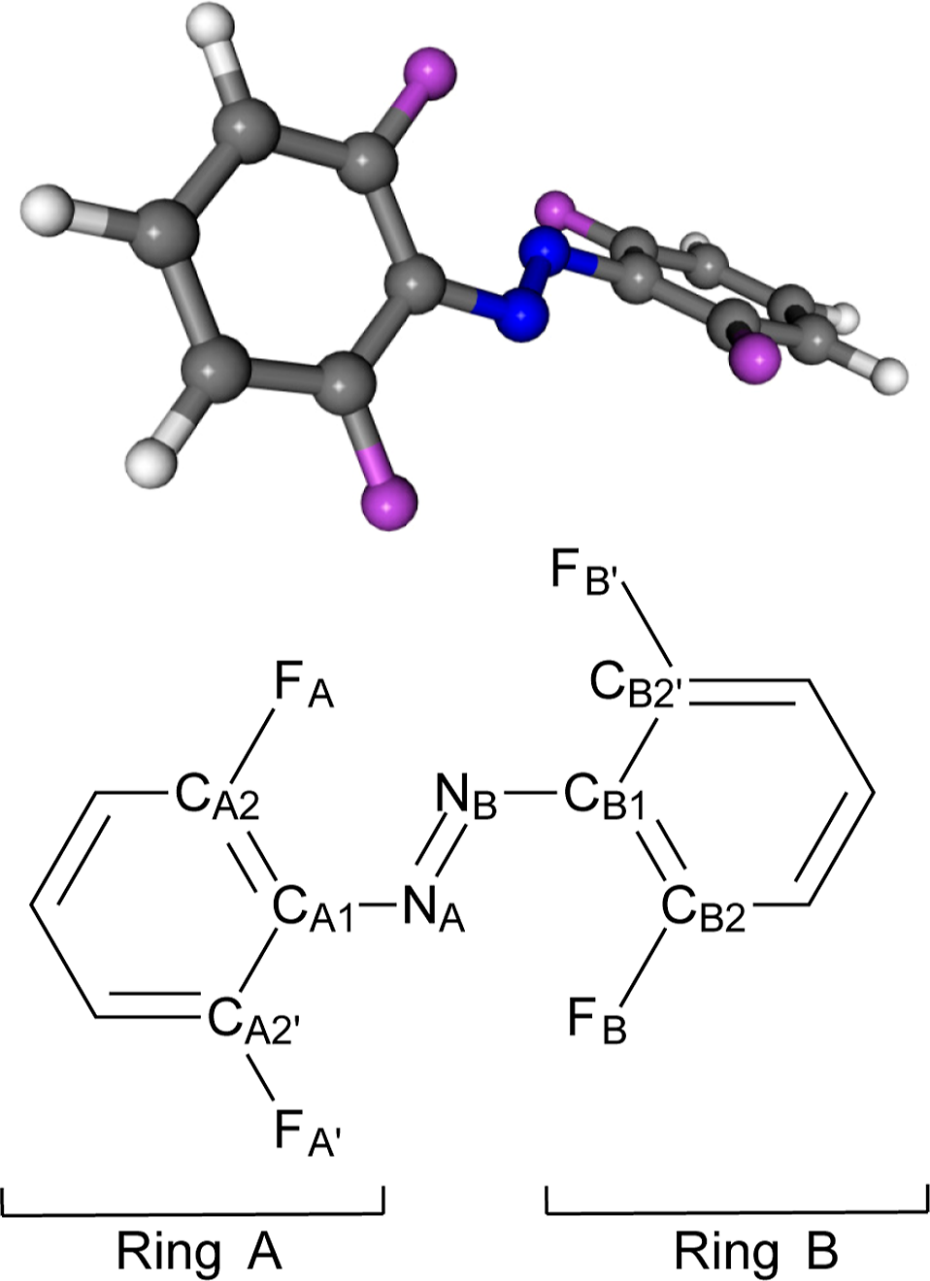
Minimum energy structure of *E*-*o*-tetrafluoroazobenzene. Top: minimum energy structure where the staggered arrangement of the two phenyl groups can be seen. Bottom: atom labels.

Vertical excitations for 1PA, 2PA, and 3PA have been determined for the above structure. We have verified that the effect of the solvent (DMSO) on the excitation energies is, in this case, small (see the Supporting Information for details). Then, to make 3PA calculations affordable, excitation energies have been computed in vacuo for 1PA, 2PA, and 3PA. Results are presented in Table 2. Excitation energies are shifted approximately 20 nm from the reported experimental absorption peaks. If we assume that the wavelength corresponding to the maximum of absorption corresponds to the Franck–Condon (vertical) transition from the potential energy minimum in the ground state, then this value represents a shift of +0.37 eV in the case of the ππ* excitation (only −0.11 eV for the nπ* excitation) which is slightly larger than the known tolerance of the CAM-B3LYP functional for excitation energies in chromophores of similar sizes to our photoswitch.^54^ The shift might also be due, in part, to the disregard of vibronic effects implicit in the identification of the vertical transition from the minimum with the maximum of absorption. The vertical excitation calculations also yield magnitudes related to the probability of the transition or indirectly to its intensity. For 1PA, the square modulus of the transition dipole moment (|μ_*fi*_|^2^) is proportional to the transition probability and is given in Table 2. For 2PA and 3PA absorption, the so-called cross sections (σ_2_ and σ_3_) are also given. For all cases, Table 2 shows that the ππ* transition is at least 1 order of magnitude brighter than the nπ* transition.

**Table 2:** Vertical Transitions from the Ground-State Minimum Energy Structure of *E*-*o*-Tetrafluoroazobenzene

		S_0_ → S_1_ (nπ*)	S_0_ → S_2_ (ππ*)
	ΔE/eV	2.60	4.54
	λmax/nm	478	273
1P	|μ_*if*_|^2^/a.u^a^	8.58 × 10^–4^	5.90 × 10^–1^
2P	σ_2_/GM^b^	4.54 × 10^–3^	6.86 × 10^–2^
3P	σ_3_/a.u^c^	1.11 × 10^+6^	2.84 × 10^+7^

a 1 au for |μ_*if*_|^2^ = 7.19 × 10^–59^ C^2^ m^2^ = 6.45 D^2^.

b 1 GM (Goeppert–Mayer) = 10^–50^ cm^4^ s photon^–1^.

c 1 au for σ_3_ = 1.28 × 10^–83^ cm^6^ s^2^ photon^–2^.

**Figure 2:**
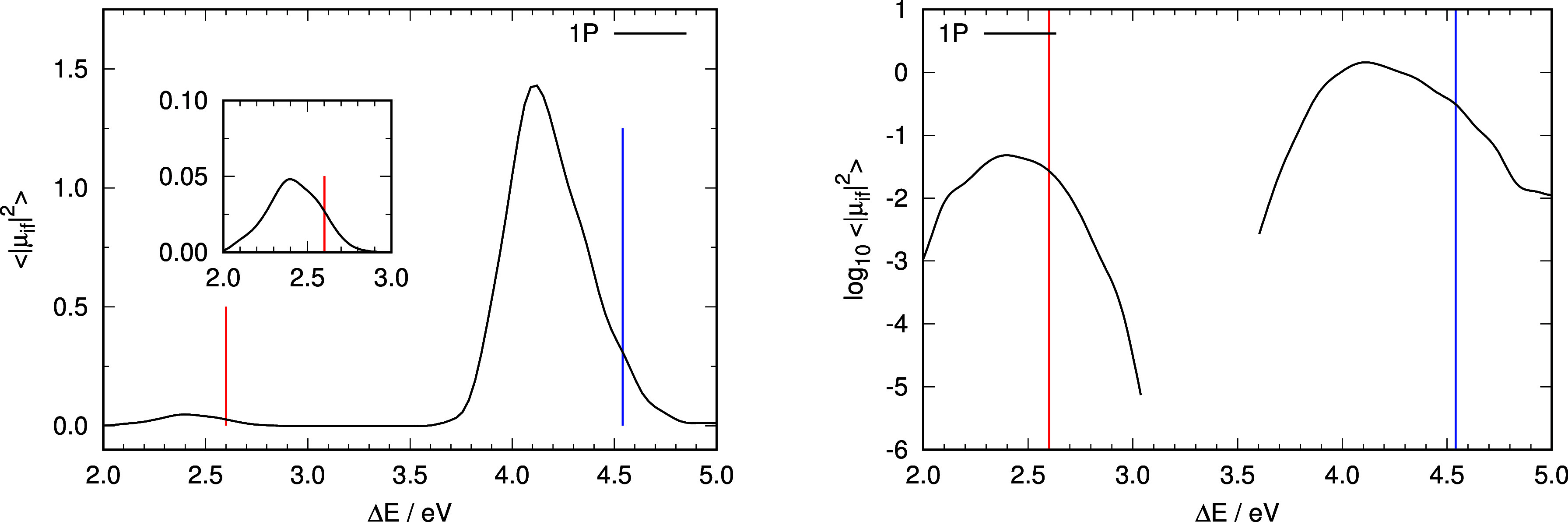
Representation of the computed absorption spectrum under 1PA conditions. Left panel: computed relative intensities of the absorption spectra under 1PA conditions. Insets represent an enlargement of the absorption spectra in the region of the nπ* excitation using the same units as the complete graph. Right panel: representation of the logarithm of the average intensities as a function of excitation energy under 1PA conditions. Vertical lines in all panels indicate vertical excitation energies from the ground-state minimum energy structure to S_1_ (nπ* state, red line) and S_2_ (ππ* state, blue line).

**Figure 3:**
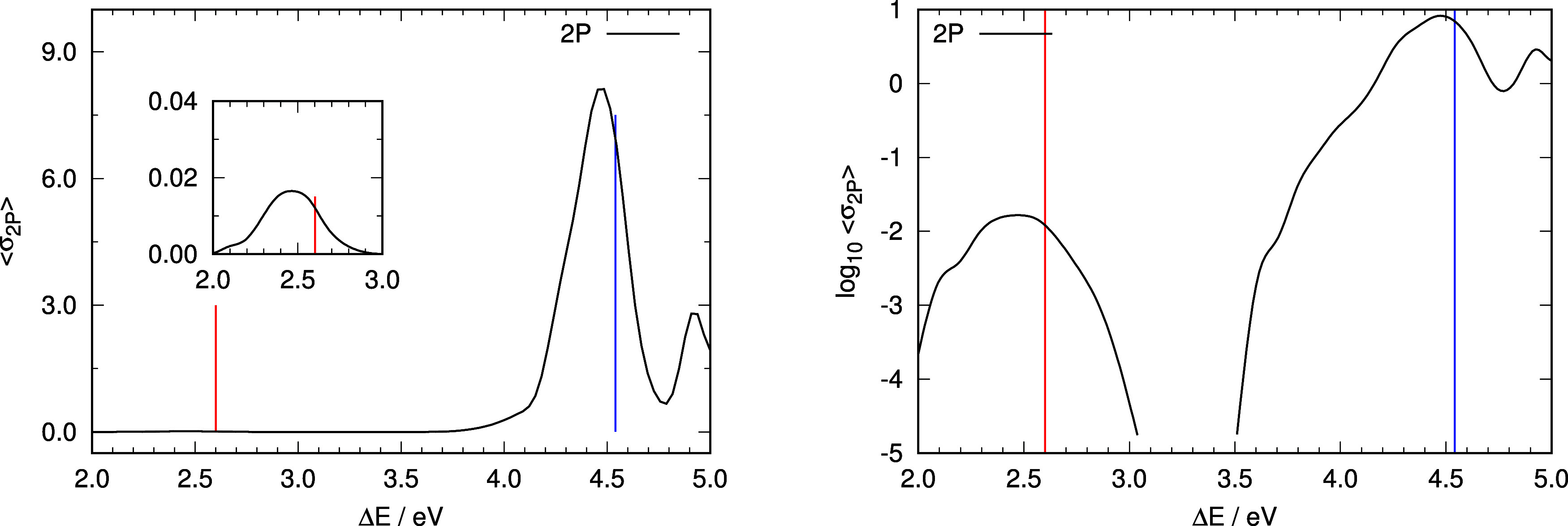
Representation of the computed absorption spectrum under 2PA conditions. Left panel: computed relative intensities of the absorption spectra under 2PA conditions. Insets represent an enlargement of the absorption spectra in the region of the nπ* excitation using the same units as the complete graph. Right panel: representation of the logarithm of the average intensities as a function of excitation energy under 2PA conditions. Vertical lines in all panels indicate vertical excitation energies from the ground-state minimum energy structure to S_1_ (nπ* state, red line) and S_2_ (ππ* state, blue line).

**Figure 4:**
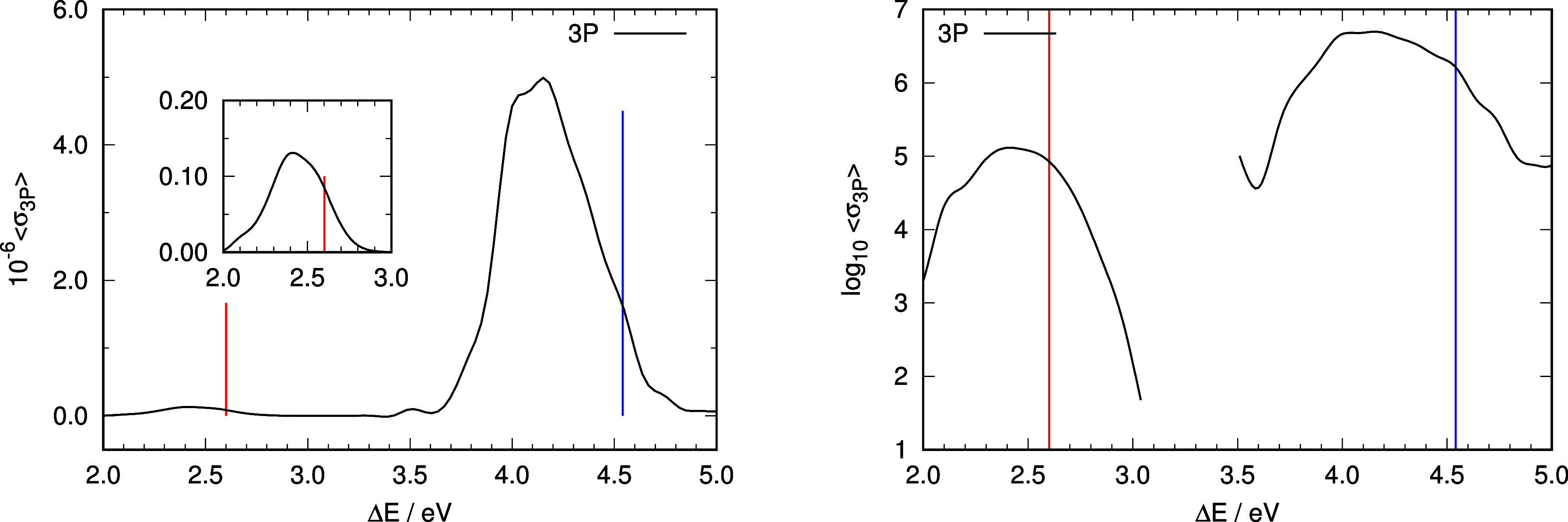
Representation of the computed absorption spectrum under 3PA conditions. Left panel: computed relative intensities of the absorption spectra under 3PA conditions. Insets represent an enlargement of the absorption spectra in the region of the nπ* excitation using the same units as the complete graph. Right panel: representation of the logarithm of the average intensities as a function of excitation energy under 3PA conditions. Vertical lines in all panels indicate vertical excitation energies from the ground-state minimum energy structure to S_1_ (nπ* state, red line) and S_2_ (ππ* state, blue line).

How well do vertical transitions from the ground-state minimum compare to the absorption spectra computed for this molecule in solution? Following the procedure described in the Methods section, a total of 30 ns of QM/MM MD simulation of a molecule of *E*-*o*-tetrafluoroazobenzene solvated in DMSO has been computed. From the resulting trajectory, we have selected 3000 uncorrelated snapshots and used them to compute the corresponding magnitude related to the intensity of absorption in 1PA, 2PA, and 3PA and used them to construct a simulation of the absorption spectra. Figure 2 shows the simulated 1PA spectrum, whereas the 2PA and 3PA spectra are shown in Figures 3 and 4. In general, vertical excitation energies from the ground-state minimum (shown as vertical lines in Figures 2–4) appear at higher energies than the respective absorption maxima: in the case of 1PA and 3PA by ∼0.4 eV for the ππ* transition and ∼0.2 eV for the nπ* transition. 2PA shows an inappreciable nπ* transition, but the band maximum of the ππ* excitation is much closer to the vertical transition from the ground-state minimum (off by 0.1 eV). The trend observed on the band maxima to be red-shifted with respect to the vertical excitation on the ground electronic state can actually be understood based on the behavior of the molecule in solution: the molecule can vibrate (anharmonically), which results in bonds that are on average longer than in the minimum energy structure. This will be further exaggerated by interaction with solvent molecules. The distortion of the geometry of the solute molecule will bring about higher energies for the ground state, and in general, a lowering of the excited electronic state energy, with an overall effect of a decrease of the excitation energy with respect to the value at the minimum energy structure.

Relative intensities of the two transitions are also not well reproduced by the values derived from the respective vertical transitions from the ground-state minimum. Right panels in Figures 2 and 4 show about one-and-a-half orders of magnitude difference between the ππ* and nπ* absorption bands in 1PA and 3PA conditions, while the difference is almost 3 orders of magnitude for 2PA (Figure 3). The ratio of intensities of the ππ* to nπ* transitions using vertical excitation data are 687 (1PA), 15 (2PA), and 16 (3PA). To compare these values with those derived from the ensemble calculations, we have computed the integral of the intensities for each band separately in 1PA, 2PA, and 3PA and then computed the ratio of intensities of the ππ* to nπ* bands. The values obtained are 35, 485, and 52 for 1PA, 2PA, and 3PA respectively.

**Figure 5:**
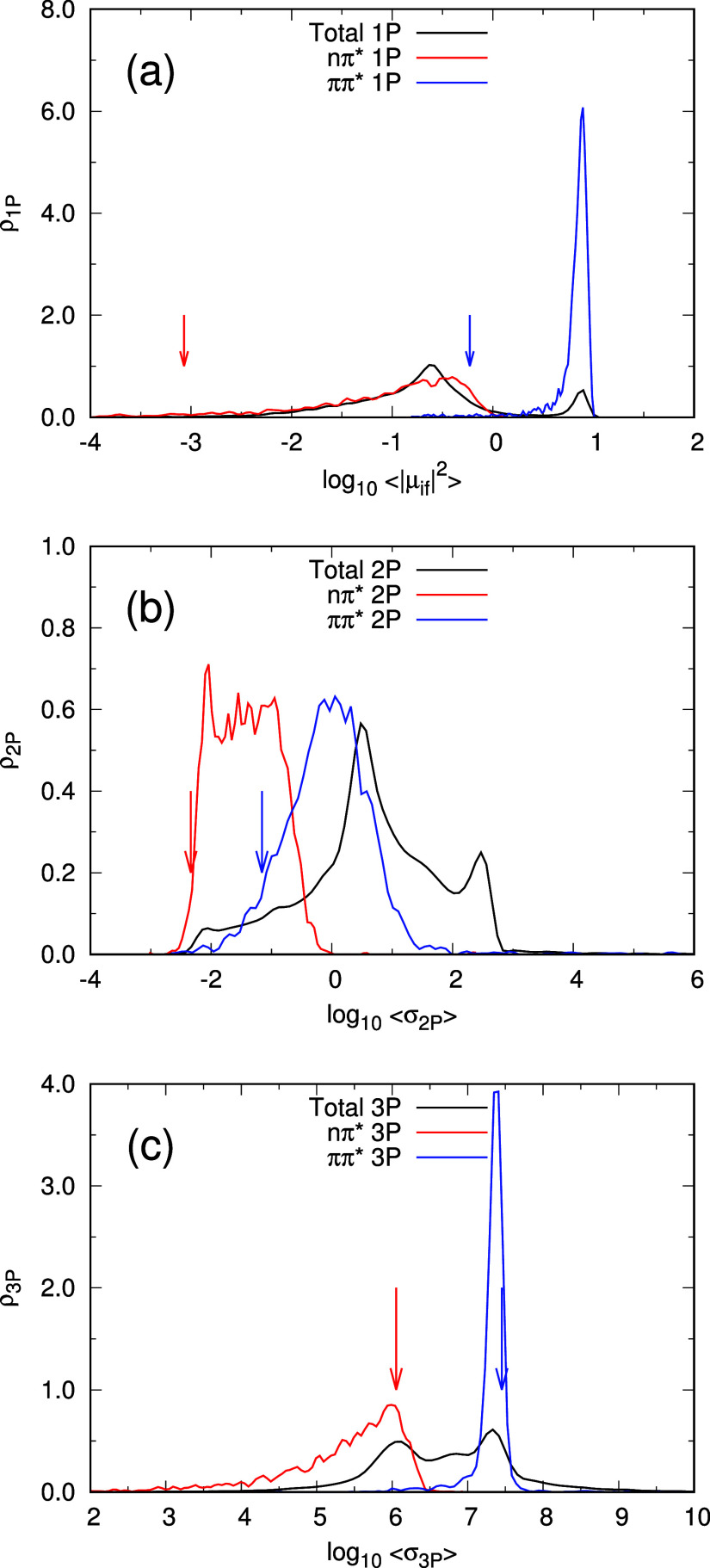
Normalized distribution of MD snapshots across the intensity-related magnitude level under 1PA (a), 2PA (b), and 3PA (c) conditions: red lines are the distribution of contributions to the nπ* excited state, blue lines represent the distribution of contributions to the ππ* excited state, and the black lines represent the global distribution combining all transitions. The red and blue arrows indicate the location of the vertical transition from the ground-state minimum to S_1_ and S_2_, respectively. Note that the abscissa axis is represented in the logarithmic scale.

Figure 5 shows the distribution of structures from the dynamics according to the relative intensity of the transitions to each excited electronic state. Except on the 3PA case [panel (c)], the contribution of the vertical transition from the ground-state minimum to each band would be among the weakest, or in other words, vertical transition from the ground-state minimum contributes very little to the actual intensity of the transition. Thus, the vertical lines in Figures 2–4 indicate the position of the vertical transitions from the ground-state minimum energy structure, but the intensity measured for the spectrum at a given energy is the result of all contributions of all vertical transitions at that energy, only one of which corresponds to the transition from the ground-state minimum (and with low probability, as can be seen in Figure 5).

It looks like the conventional recipe of estimating the absorption properties of a molecule via determination of the vertical transition at the minimum energy structure is not adequate in all cases. Table 3 shows quantitative data regarding the position of the computed absorption band maxima and quantification of their size as well as their position. The absorption bands in all spectra are quite broad, with full width at half-maximum (fwhm) values in the range 0.32–0.52 eV, or in terms of wavelength between 20 and 146 nm, depending on the type of spectroscopy and excited state involved. The actual maxima found are always red-shifted for both states with respect to the vertical excitation from the ground-state minimum energy structure by up to −0.39 eV (or bathochromically shifted by up to 36 nm). In fact, the excitation energies from the ground-state minimum energy structure for both states are practically outside of the fraction of the absorption band defined by the fwhm value in many cases. Except for the 2PA ππ* excitation, it is clear that vertical excitation does not contribute significantly to it. In particular, the spread of intensities and excitation energies shown in Figures 2–4 indicates that for molecules with a certain fluxionality like this, the minimum energy structure is not a good representative of the many structures that the system evolves along the dynamics.

**Table 3:** Computed Absorption Band Maxima, Band Size, and Shift of Band Maxima from Vertical Transition from Minimum Energy Structure

transition	Δ*E*^a^ (eV)	λ^a^ (nm)	Emin–Emax^b^ (eV)	fwhm^c^		peak shift^d^	
				(eV)	(nm)	(eV)	(nm)
1PA: nπ*	2.41	514	2.25–2.60	0.35	74	–0.19	+36
2PA: nπ*	2.46	504	2.27–2.65	0.38	78	–0.14	+26
3PA: nπ*	2.41	514	2.25–2.64	0.39	81	–0.19	+36
1PA: ππ*	4.11	302	3.94–4.38	0.44	32	–0.43	+29
2PA: ππ*	4.47	277	4.29–4.61	0.32	20	–0.07	+4
3PA: ππ*	4.15	299	3.91–4.43	0.52	146	–0.39	+26

a Maxima of the computed bands.

b Limits of the absorption band inside the full width at half-maximum range.

c Width of the absorption band given as the full width at half-maximum.

d Shift of the computed band maxima with respect to the vertical transition from the ground-state minimum (q.v. Table 2).

**Figure 6:**
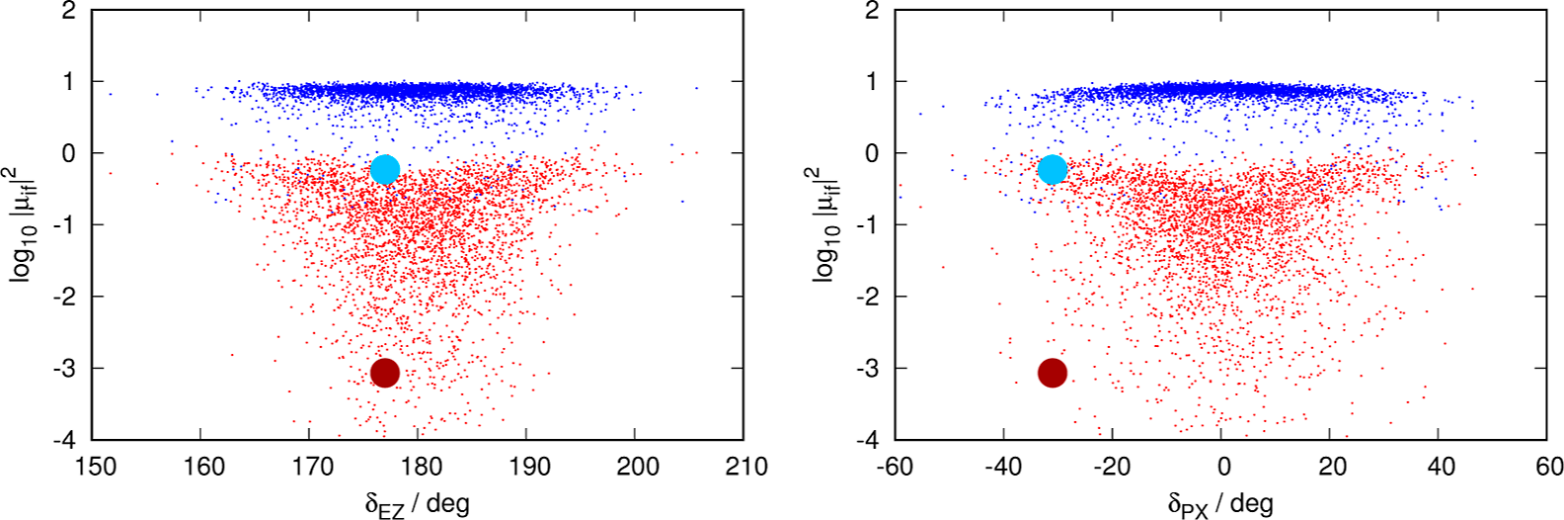
Distribution of contributions to the 1PA spectrum classified by each structure’s transition intensity and geometry. Left panel: distribution as a function of dihedral δ_EZ_ of each structure. Right panel: distribution as a function of δ_PX_ (dihedral δ_PA_ or δ_PB_, whichever has the smallest absolute value) of each structure. Each snapshot of the dynamics contributes one red dot and one blue dot to each graph: red and blue dots identify the nπ* and the ππ* transitions, respectively. The data for the vertical transition from the ground-state minimum are presented as a big red point (nπ*) and big blue point (ππ*). Note that the ordinate axis (intensity) is in the logarithmic scale.

**Figure 7:**
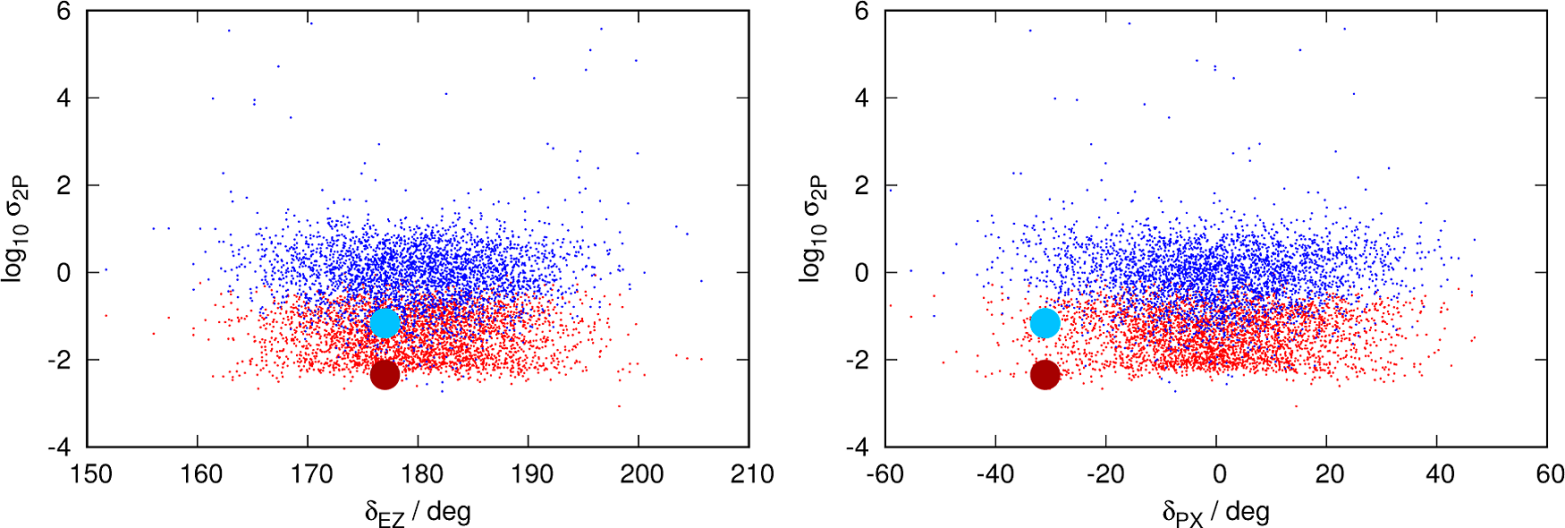
Distribution of contributions to 2PA spectrum classified by each structure’s transition intensity and geometry. Left panel: distribution as a function of dihedral δ_EZ_ of each structure. Right panel: distribution as a function of δ_PX_ (dihedral δ_PA_ or δ_PB_, whichever has the smallest absolute value) of each structure. Each snapshot of the dynamics contributes one red dot and one blue dot to each graph: red and blue dots identify the nπ* and the ππ* transitions, respectively. The data for the vertical transition from the ground-state minimum are presented as a big red point (nπ*) and big blue point (ππ*). Note that the ordinate axis (intensity) is in the logarithmic scale.

**Figure 8:**
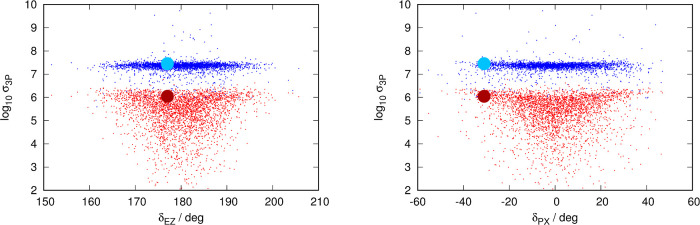
Distribution of contributions to the 3PA spectrum classified by each structure’s transition intensity and geometry. Left panel: distribution as a function of dihedral δ_EZ_ of each structure. Right panel: distribution as a function of δ_PX_ (dihedral δ_PA_ or δ_PB_, whichever has the smallest absolute value) of each structure. Each snapshot of the dynamics contributes one red dot and one blue dot to each graph: red and blue dots identify the nπ* and the ππ* transitions, respectively. The data for the vertical transition from the ground-state minimum are presented as a big red point (nπ*) and big blue point (ππ*). Note that the ordinate axis (intensity) is in the logarithmic scale.

An important characteristic of this molecule is the possibility of having an extended π system when the coplanarity of the phenyl rings and the N=N moiety is achieved in practice. How often this happens and how close to planarity the system comes depends on the dynamics and mainly on the fluxionality of the photoswitch. It is this what determines the main features of the absorption spectra. We have analyzed the snapshots used to build the spectra correlating the magnitude related to transition probability in each case with the values of the dihedral angles that define the coplanarity. Results for 1PA are presented in Figure 6, for 2PA in Figure 7, and for 3PA in Figure 8.

Figures 6–8 reveal a striking similarity between the plots describing the distribution of structures for 1PA and 3PA, with those of 2PA being clearly different. This was already visible in the spectra (Figures 2–4), where the nπ* was only visible for 1PA and 3PA, where the position and structure of the ππ* band are very similar, and different from the 2PA case.

In the 1PA/3PA cases, the contributions to the ππ* band are tightly clustered as far as the intensity parameter is concerned with a spread of about half an order of magnitude and almost no structures with weak contributions. In contrast, contributions to the nπ* band are substantially more spread out, presenting a distribution resembling an inverted triangle: a substantial number of structures have very weak transitions (10^4^ times weaker than the most intense contributions), especially at values close to δ_EZ_ = 180 and δ_PX_ = 0°. It can also be seen that for these two spectroscopic techniques, there is a “gap” of 1 order of magnitude between the top contributions to the ππ* and nπ* bands. 2PA sets itself apart from the other two spectroscopic techniques. The contributions to both bands are distributed evenly, with respect to both dihedral angles.

Vertical transitions from the ground-state minimum, on the other hand, are proven to give poor estimates about the intensity of the transition. 1PA and 2PA transitions, as derived from the vertical transitions from the ground-state minimum, rank among the weakest excitations sampled by the dynamics.

## 4 Conclusions

We conclude this study by highlighting that, despite the fact that photoswitch *E*-*o*-tetrafluoroazobenzene is not centrosymmetric, a marked similarity pervades different aspects of its 1PA and 3PA spectroscopies, with 2PA being set clearly apart as expected in centrosymmetric molecules. Enforcing planarity (and because of this, centrosymmetric character) and analyzing how the transition probabilities change when planarity is lifted reveal two interesting facts. First, we have observed that 1PA, 2PA, and 3PA transition probabilities behave in exactly opposite ways for the nπ* and ππ* electronic excited states. Second, we have seen that 1PA and 3PA transition probabilities show the same behavior when leaving planarity, a behavior that is opposite to that shown for 2PA. This means that while 2PA absorption cross sections to access the nπ* remain approximately constant when the photoswitch leaves planarity, 1PA and 3PA transition probabilities display a steep increase for the same state. The behaviors change exactly to the opposite when access is considered to state ππ*.

Simulated absorption bands have been compared with the predicted energy and wavelength of maximum absorption following the conventional approach based on vertical excitation departing from the ground electronic state minimum energy structure. This conventional approach does not provide a clear way of estimating the extent of the absorption bands. We have found that the bands are broad, with fwhm values between 0.32 and 0.52 eV (20–146 nm) depending on the state and type of spectroscopy being considered. The actual maxima found are always red-shifted for both electronic states with respect to the latter by an amount of up to −0.39 eV (or bathochromically shifted by up to 36 nm). It is also significant that the usual approach to determine the maximum of the absorption band renders the same values for 1PA, 2PA, and 3PA. However, our results indicate that there is a certain variation in the value of the excitation energies, which again sets 2PA apart (about 0.35 eV higher in energy) from the 1PA and 3PA spectroscopies.

## Supporting Information Available

Study of the effect of the basis set size on the excitation energies and the transition probabilities and study of the effect of the presence or absence of solvent on the energy of the transitions (PDF)
