# Supplementary material for: Effect of Leaving Centrosymmetric Character on Spectral Properties in Mono-, Bi-, and Triphotonic Absorption Spectroscopies
Source: ACS Omega. 2024 Sep 25;9(40):41968–77. doi: 10.1021/acsomega.4c06922 (PMC11465556; doi:10.1021/acsomega.4c06922)
Supplement: Supplementary file 1 — ao4c06922_si_001.pdf [file ao4c06922_si_001.pdf]

Supporting Information for:

Effect of Leaving Centrosymmetric Character on Spectral Properties in Mono-, Bi- and Triphotonic Absorption Spectroscopies

Ricard Gelabert*, Miquel Moreno, and José M. Lluch

Departament de Química, and Institut de Biotecnologia i de Biomedicina, Universitat Autònoma de Barcelona, 08193 Bellaterra, Barcelona, Spain

Contents

1 Deciding the Level of Calculation for Electronic Structure S2

1.1 Assessment of the Effect of the Size of the Basis Set S2

1.2 Comparison of Excitation Energies With and Without Solvent S2

1 Deciding the Level of Calculation for Electronic Structure

The goal of this work was to compute three absorption spectra combining the transition probabilities under 1PA, 2PA and 3PA conditions of a large set of structures. Particularly for 3PA, the cost increases very quickly with the size of the basis set, and because a converged spectrum requires the calculation on many contributing structures, it is a critical decision. Aside from this, the Dalton software suite we use for computation of the 2PA and 3PA excitations cannot in this later case introduce the effect of a polarizable continuum to simulate the effect of the solvent. Because of this, we have analyzed what is the effect of the basis set as well as the effect of introducing or not the solvent exclusively on the excitation energies.

1.1 Assessment of the Effect of the Size of the Basis Set

We have in the past, in works limited to 1PA spectroscopy, used the triple-ζ 6-311+G(d,p) basis set routinely. This basis set makes 2PA, and especially 3PA calculations very expensive. We have tested the double-ζ 6-31G(d,p) basis set as a test of the effect on excitation energies and the quantities related to the transition probability on the structure of the minimum, for the two excited states. The calculations have been performed on the geometry of the minimum energy structure minimized at 6-311+G(d,p) level in DMSO (via polarizable continuum). Excitation energies and transition probability-related magnitudes are calculated *in vacuo* and shown in Table S1 for the 6-31G(d) and in Table S2 for the 6-311+G(d,p) basis sets.

Results shown indicate that excitation energies agree within 0.1 eV in the two basis sets. Transition probability-related magnitudes are very similar for 2PA and 3PA. In the case of 1PA, values with triple-ζ basis set are about one order of magnitude larger for the $\pi\pi^*$ excitation.

1.2 Comparison of Excitation Energies With and Without Solvent

Current DALTON implementation cannot perform 3PA calculations when solvent is implicit (CPCM) and thus vertical excitation energies and transition probability-related magnitudes have to be evaluated in vacuo. To assess the magnitude of the error incurred, we have evaluated on the structure of the minimum the excitation energies and transition probability-related

		$S_0 \rightarrow S_1$ ($n\pi^*$)	$S_0 \rightarrow S_2$ ($\pi\pi^*$)
	ΔE / eV	2.60	4.54
	$\lambda_{\max}$ / nm	478	273
1P	$\|\mu_{if}\|^2$ / a.u.^{a}	2.12×10^{-1}	5.90×10^{-1}
2P	σ_2 / GM^b	4.54×10^{-3}	6.86×10^{-2}
3P	σ_3 / a.u.^{c}	$1.11 \times 10^{+6}$	$2.84 \times 10^{+7}$

^{a} 1 a.u. for $|\mu_{if}|^2 = 7.19 \times 10^{-59}$ C^2 m^2 = 6.45 D^2.
^{b} 1 GM (Goeppert-Mayer) = 10^{-50} cm^4 s photon^{-1}.
^{c} 1 a.u. for $\sigma_3 = 1.28 \times 10^{-83}$ cm^6 s^2 photon^{-2}.

Table S1: Vertical Transitions of *E*-*o*-tetrafluoroazobenzene with 6-31G(d) Basis Set computed *in vacuo*. The structure corresponds to the minimum energy structure in the ground state computed with the 6-311+G(d,p) basis set and including DMSO as solvent using CPCM.

		$S_0 \rightarrow S_1$ ($n\pi^*$)	$S_0 \rightarrow S_2$ ($\pi\pi^*$)
	ΔE / eV	2.61	4.44
	$\lambda_{\max}$ / nm	475	279
1P	$\|\mu_{if}\|^2$ / a.u.^{a}	5.10×10^{-1}	$7.13 \times 10^{+0}$
2P	σ_2 / GM^b	2.97×10^{-3}	5.41×10^{-2}
3P	σ_3 / a.u.^{c}	$1.41 \times 10^{+6}$	$3.51 \times 10^{+7}$

^{a} 1 a.u. for $|\mu_{if}|^2 = 7.19 \times 10^{-59}$ C^2 m^2 = 6.45 D^2.
^{b} 1 GM (Goeppert-Mayer) = 10^{-50} cm^4 s photon^{-1}.
^{c} 1 a.u. for $\sigma_3 = 1.28 \times 10^{-83}$ cm^6 s^2 photon^{-2}.

Table S2: Vertical Transitions of *E*-*o*-tetrafluoroazobenzene with 6-311+G(d,p) Basis Set computed *in vacuo*. The structure corresponds to the minimum energy structure in the ground state computed with the 6-311+G(d,p) basis set and including DMSO as solvent using CPCM.

magnitudes in vacuum. Results are presented in Table S3, and meaningful comparisons should be made with Table S1.

Excitation energies vary a little, with the most important change being a bathochromic shift of 0.32 eV for the $\pi\pi^*$ transition, roughly equivalent to 30 nm at this energy.

We note that computation of 3PA cross sections including solvent as

		$S_0 \rightarrow S_1$ $(n\pi^*)$	$S_0 \rightarrow S_2$ $(\pi\pi^*)$
	ΔE / eV	2.64	4.22
	λ_{max} / nm	478	273
1P	$\|\mu_{if}\|^2$ / a.u.^{a}	9.72×10^{-1}	$1.01 \times 10^{+1}$
2P	$\sigma_{2\text{P}}$ / GM^b	1.54×10^{-2}	6.06×10^{-2}

^{a} 1 a.u. for $|\mu_{if}|^2 = 7.19 \times 10^{-59}$ C^2 m^2 = 6.45 D^2.

^{b} 1 GM (Goeppert-Mayer) = 10^{-50} cm^4 s photon^{-1}

Table S3: Vertical Transitions of E-o-tetrafluoroazobenzene with 6-31G(d) Basis Set and solvent introduced via CPCM (DMSO). The structure corresponds to the minimum energy structure in the ground state computed with the 6-311+G(d,p) basis set and including DMSO as solvent using CPCM.

polarizable continuum is possible using external software libraries.[1,2]

References

[1] Di Remigio, R.; Beerepoot, M. T. P.; Cornaton, Y.; Ringholm, M.; Steindal, A. H.; Ruud, K.; Frediani, L. Open-ended formulation of self-consistent field response theory with the polarizable continuum model for solvation. *Phys. Chem. Chem. Phys.* **2017**, *19*, 366 – 379.

[2] `PCMSolver`, an open-source library for the polarizable continuum mode electrostatic problem, written by R. Di Remigio, L. Frediani and contributors (see http://pcmsolver.readthedocs.io/).
